# Oxidation of Isoeugenol by Salen Complexes with Bulky Substituents

**DOI:** 10.3390/ijms11030912

**Published:** 2010-03-04

**Authors:** Anika Salanti, Marco Orlandi, Eeva-Liisa Tolppa, Luca Zoia

**Affiliations:** Dipartimento di Scienze dell’Ambiente e del Territorio, Università degli Studi di Milano-Bicocca, Piazza della Scienza, 1, 20126, Milano, Italy; E-Mails: marco.orlandi@unimib.it (M.O.); eeva-liisa.tolppa@unimib.it (E.-L.T.); luca.zoia@unimib.it (L.Z.)

**Keywords:** salen, isoeugenol, dehydrogenative polymerization, GPC, NMR

## Abstract

The catalytic properties of bulky water-soluble salen complexes in the oxidation of isoeugenol (2-methoxy-4-(1-propenyl) phenol) have been investigated in aqueous ethanol solutions in order to obtain a mixture of polymeric compounds through dehydrogenative polymerization. The average molecular weight of dehydrogenated polymers (DHPs) was monitored by GPC and correlated to reaction conditions such as time, concentration of substrate, concentration of catalyst, type of oxidation agent, *etc*. The DHP synthesized by adopting the best reaction conditions was characterized by different analytical techniques (GPC, ^13^C-NMR, ^31^P-NMR and LC-MS) to elucidate its structure. The lignin-like polymer resulting from isoeugenol radical coupling possesses valuable biological activity and finds applications in a variety of fields, such as packaging industry and cultural heritage conservation.

## 1. Introduction

Plant polyphenols constitute an important source of renewable carbon in the biosphere. Although the oxidation of lignocellulosic materials has been extensively studied in the last decades, our knowledge of the enzymes participating in the biosynthesis and in the oxidative process is still limited. The majority of these enzymes (namely lignin peroxidases, manganese peroxidases and laccases) are difficult to isolate in pure form and, moreover, mediators such as Mn ions and veratryl alcohol, also seem to be involved in the reaction mechanism. Therefore, more readily obtainable and stronger oxidizing catalyst such as salens, porphyrins and phthalocyanines, have been commonly used [1,2]. Salen complexes are a major class of coordination compounds, which have been exploited since 1933 to catalyze a wide variety of reactions [3]. The most of these reactions are oxidations of organic substrates, based on the use of oxidants such as molecular oxygen and hydrogen peroxide. The synthesis of either water or organic solvent-soluble salen complexes is simple, easy and economic. Metal salen compounds have been investigated as catalysts in several different reactions, for example epoxidation of olefins [4] and oxidation of sulphides to sulphoxides [5]. The most valuable property of salen complexes lies in their modulatory nature: the ligand structure, as well as the coordinated metal ion, can be easily varied to modify their hydrophilicity to match the end-use application [6]. Another important reaction of salen complexes is the single electron oxidation of phenols to produce phenoxy radicals, and then, by radical coupling, dehydrogenative polymerization products (DHPs) [7–9]. The reaction, when applied to isoeugenol, results in a polymer with a lignin-like structure but it is more hydrophobic [10–12].

Isoeugenol is a clear to pale yellow oily liquid present in certain essential oils especially in clove oil and cinnamon. It is used in manufacturing perfumeries, flavourings, essential oils, medicine (local antiseptic and analgesic) and possesses important antioxidant and anti-bacteria activity [13]. Dehydrogenated polymers were previously synthesized from coniferyl alcohol and isoeugenol by means of an oxidative coupling approach choosing inorganic salts [14] or inorganic oxides [15] as one-electron oxidants. The DHPs obtained from isoeugenol radical coupling has been successfully applied in the consolidation of waterlogged woods [16] and in the modification of cellulose packaging to confer antimicrobial properties [17], where higher molecular weights were assumed to be the most efficient. In this work, DHPs from isoeugenol have been synthetized and their molecular weight were related to different reaction conditions in order to identify a set of requisites which provide the highest degree of polymerization. Subsequently, the chemical features of the optimized product, *i.e.*, the DHP characterized by the highest molecular weight, were elucidated by spectroscopic techniques. Furthermore, the antioxidant properties of the obtained isoeugenol polymer were investigated.

## 2. Results and Discussion

### 2.1. Synthesis of Sulfosalen and Phosphosalen

**Scheme 1.**
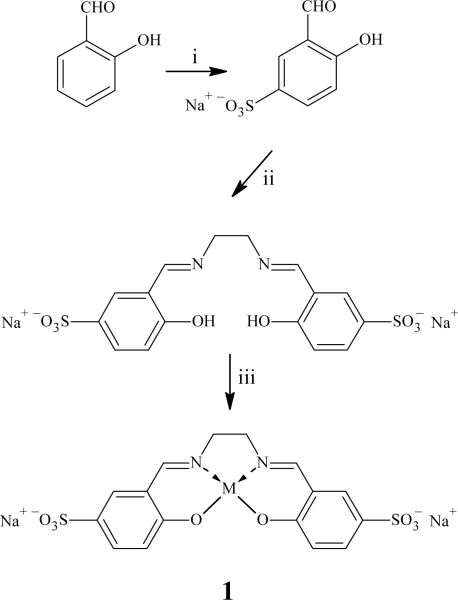
Synthesis of SulfoSalen (SS). Reagents and conditions: i) H_2_SO_4_ at RT for 24 h, H_2_O and NaHCO_3_; ii) ethylenediamine, EtOH at 78 °C for 4 h; iii) M(OAc)_n_ or MCl_n_, EtOH at 78 °C for 3 h. M = Cu, Co, Mn, Fe.

**Scheme 2.**
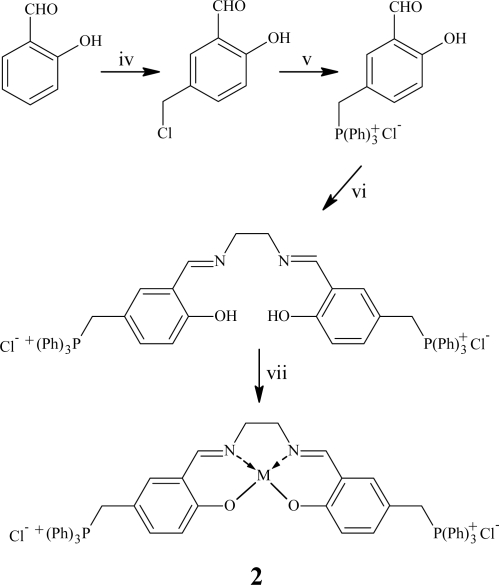
Synthesis of PhosphoSalen (PS). *Reagents and conditions:* iv) H_2_CO and HCl 37% at RT for 3.5 h; v) P(Ph)_3_ in benzene at reflux for 1 h; vi) ethylenediamine, EtOH at 78 °C for 4 h; vii- M(OAc)_n_ or MCl_n_, EtOH at 78 °C for 3 h. M = Cu, Co

In order to investigate the catalytic properties of salen complexes a new set of water-soluble salen were synthetized following Schemes 1 and 2.

### 2.2. Optimization of Polymerization Conditions

**Figure 1.**
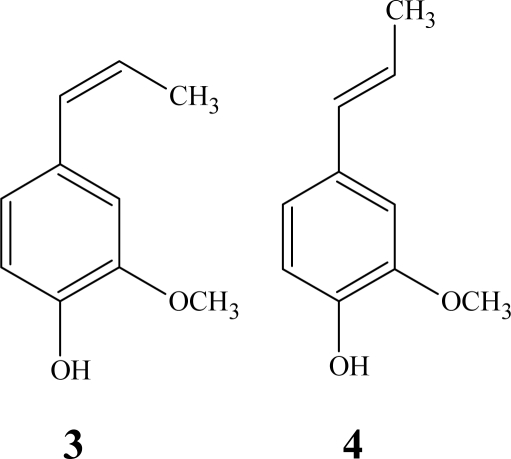
Isoeugenol: 2-methoxy-4-[(*Z*)-1-propenyl] phenol (**3**) and 2-methoxy-4-[(*E*)-1-propenyl] phenol (**4**).

The catalytic properties of water-soluble salen complexes were investigated by changing the metal ions in the active centre and varying the reaction conditions in order to optimize the polymerization degree of isoeugenol (2-methoxy-4-(1-propenyl) phenol) (Figure 1). The polymerization degree was evaluated by GPC analyses.

The first reaction followed was the oxidation of isoeugenol in a 1:1 water-ethanol solution with different catalysts. Ethanol is required as a cosolvent as isoeugenol is not water-soluble. Two types of bulky salen complexes were used: the SulphoSalen (SS, **1**) and the PhosphoSalen (PS, **2**). The effectiveness of four different metal ions acting as the active center were tested: Manganese (Mn), Iron (Fe), Cobalt (Co) and Copper (Cu) for Sulphosalen complexes (**1**); Copper (Cu) and Cobalt (Co) for Phosphosalen complexes (**2**). During the reaction, isoeugenol consumption was monitored by TLC. The reaction was carried out for 48 hours at room temperature under stirring. In these conditions the main products are isoeugenol oligomers generated by coupling reactions of phenoxy radicals. The concentration of isoeugenol in the solution was equal to 1% w/v and the concentration of catalyst 0.1% w/v. At the end of reaction, the molecular weight of isoeugenol oligomers were determined on extracted products by GPC analysis (Table 1).

**Figure 2.**
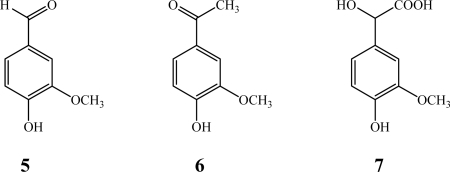
Main oxidation products of isoeugenol: vanillin (**5**), acetovanillone (**6**) and vanillylmandelic acid (**7**), as detected by GC-MS analysis.

Mn- and Fe-Salen catalyzed oxidations are known to occur through metal-oxo complexes in the presence of hydrogen peroxide, while Co- and Cu- complexes activate dioxygen forming a 1:1 metal to oxygen superoxo or a 2:1 metal to oxygen peroxo complexes as the active oxidizing species. As reported in Table 1, Iron, Manganese and Copper complexes were activated by the presence of H_2_O_2_, while Cobalt and Copper complexes were exposed to a slight overpressure of molecular oxygen (O_2_). According to literature data [7] Copper complexes (CuSS and CuPS) were found to be more active in the presence of H_2_O_2_ and led to DHPs with higher molecular weights. The obtained products were mainly oligomeric compounds with a low amount (maximum 5% w/w) of monomeric side-chain oxidation by-products. These oxidation products were characterized by GC-MS analysis, and consist mainly in vanillin (3-methoxy-4-hydroxybenzaldehyde, **5**), acetovanillone (1-(3-methoxy-4-hydroxyphenyl)-ethanone, **6**) and vanillylmandelic acid (hydroxy-(3-hydroxy-4-methoxyphenyl) acetic acid, **7**) (Figure 2).

These preliminary GPC data showed that Cu(SS) catalyzes the formation of polymers with the highest M_n_ and M_w_ in the presence of H_2_O_2_ as the oxidant. Afterwards, the effect of pH on the polymerization reaction catalyzed by Cu(SS) was taken into account monitoring the M_n_ and M_w_ values of DHPs synthesized into different buffer solution. As shown in Table 2, the molecular weight of the isoeugenol DHP was lowest at pH 4.5 and greatest at pH 9. An increased activity of Sulphosalen at higher pH value was also reported by Sippola [18].

Another parameter influencing DHPs structure is the monomer addition rate to the reaction mixture. The reagent could be added either slowly and continuatively (Zutropf method ZT) or by means of an unique, batch-mode addition (Zuluaf method ZL). Literature data report a lower molecular weight for Zuluaf DHPs, which are assumed to precipitate faster than Zutropf polymers [19]. Differences in the products composition are presumably related to the concentration of radicals. The aim of the present work is to point out changes in DHPs molecular weight distribution as the monomer addition rate is varied. Therefore, potential differences in intermonomeric linkages type and distribution have not been taking into account. In these experiments a slow rate addition was achieved by means of a step-wise approach.

According to literature data, GPC analyses showed an higher molecular weight for Zutropf DHPs (Table 3) [19].

The polymerization degree was then optimized by changing the catalyst (CuSS) concentration and the amount of isoeugenol, expressed as w/v% respect to water-ethanol solution. GPC results are reported in Table 4. A correlation between the catalyst concentration and the average molecular weight of the DHPs was observed: a rise from 0.2% up to 2% in the amount of catalyst allowed for a doubling of M_w_. Otherwise, when the same amount of catalyst was used, a straightforward relationship between isoeugenol concentration and M_w_ were not identified as the polymerization degree did not seem to be greatly affected by a monomer percentage above 1%.

Two additional parameters play a crucial role in the dehydrogenative polymerization: amount and addition rate of the oxidant into the reaction medium. For this reason, differences in the DHPs molecular weight distribution at different concentrations and at different addition rates of H_2_O_2_ were investigated. The concentration of H_2_O_2_ was expressed as an overall molar ratio between H_2_O_2_ and isoeugenol while the step-wise addition of the oxidant was carried out at regular intervals (four aliquots: one addition in the morning and one addition in the afternoon covering a reaction period of about 48 hours. Eight aliquots: four additions per day, every two hours, over two eight-hour working day. Sixteen aliquots: eight additions per day, every hour, over two eight-hour working day). Results are reported in Table 5.

Statistical analyses performed by means of the factorial ANOVA point out which parameters may affect the DHP molecular weight. It seems that both of the parameters influence the molecular weight distribution (P-value 0.01 and 0.11 for oxidant concentration and velocity of addition respectively), with the contribution of the oxidant concentration as the most important. Moreover, it is possible to observe an important interaction effect between the parameters (P-value 0.76). The best result in terms of M_n_ and M_w_ was accomplished for a H_2_O_2_/isoeugenol molar ratio equal to 10 and a relatively fast addition rate (4 different aliquots). Differences in molecular weights are presumably related to different concentrations of the monomeric phenoxy radicals generated during the reaction.

### 2.3. Polymer Characterization

**Figure 3.**
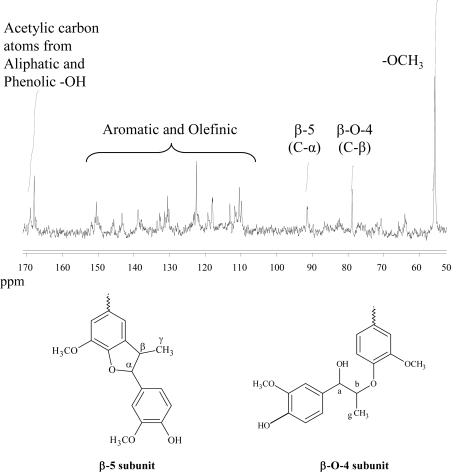
^13^C-NMR spectrum of the acetylated isoeugenol DHP and chemical structure of principal intermonomeric linkages detected (β-5, β-*O*-4).

The DHP synthesized by adopting the best reaction conditions (isoeugenol 1% w/v, CuSS as catalyst 0.2% w/v, pH 9, H_2_O_2_ as oxidant, molar ratio H_2_O_2_:isoeugenol 10:1 added in four aliquots, 48 h reaction time) was characterized by means of a range of analytical techniques: GPC, ^13^C-NMR, ^31^P-NMR and LC-MS. Gel permeation chromatography provided information about the molecular weight distribution of the product, expressed in terms of M_n_ and M_w_. Results showed that the DHP had a number-average molecular weight (M_n_) of about 2,500 g/mol and a weight-average molecular weight (M_w_) of about 8,000 g/mol. Therefore, the number of monomer units constituting the polymer was about 15, assuming 164 g/mol the molecular weight of the repeating unit. ^13^C-NMR was performed on the acetylated isoeugenol polymer [20]. The spectrum (Figure 3) showed the characteristic signals of intermonomeric bonds correlated to the β-carbon and the α-carbon of β-*O*-4 and β-5 moieties respectively [12,21]. In the range between 110–150 ppm fall many broad and partially unresolved peaks related to aromatic and olefinic carbons, as expected on the basis of the complex structure of the dehydrogenated polymer. The area comprised between 167 and 170 ppm enclose two peaks, assigned to the acetylic carbon atom connected to hydroxyl groups (phenols 167–169 ppm and secondary alcohol 169–170 ppm). Moreover, an intense signal originated by methoxylic carbon atoms is found at about 55 ppm.

**Figure 4.**
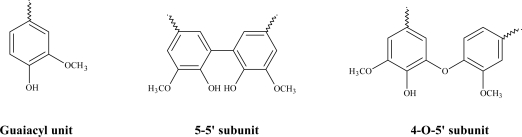
Phenolic subunit detected by ^31^P-NMR analysis (guaiacyl, 4-*O*-5’, 5-5’).

^31^P-NMR analysis was performed on the phosphytylated isoeugenol polymer in order to quantify labile -OH groups (different phenols, aliphatic hydroxyls and carboxylic acid) on the polymer [22]. The acquired spectra showed a signal related to aliphatic -OH (0.60 mmol/g, 147–150 pm), due to the presence of secondary alcohol originated from β-*O*-4 linkages. This result is in agreement with the result observed after the ^13^C-NMR analysis. The absence of primary aliphatic alcohol is associated to the structure of isoeugenol, which does not posses any hydroxyl functionality at the γ position. A slight side-chain oxidation is confirmed by the presence of a limited amount of carboxylic acid (0.03 mmol/g, 134–135 ppm). By means of ^31^P-NMR spectroscopy it is also possible to detect a moderate amount of condensed phenols, related to 5-5’ and 4-*O*-5’ phenolic structures (0.11 mmol/g, 143–145 ppm), and a large content of non-condensed guaiacyl units (0.99 mmol/g, 140–141 pm) (Figure 4).

**Figure 5.**
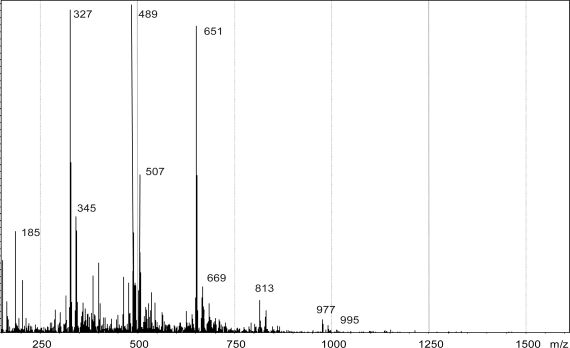
LC-MS spectrum of isoeugenol DHP.

Further confirmation on the DHP structure were obtained by LC-MS analysis. This spectroscopic technique, when soft ionization procedures are applied, act as a powerful tool in lignin investigation [23,24]. The LC-MS spectrum of isoeugenol oxidative coupling product is reported in Figure 5.

Polymerization clusters are distinctly recognizable and regularly occur every 163 amu along with patterns located at +18 amu. Regular raising mass of 163 amu could be regarded as progressive radical couplings resulting in the formation of β-5 or condensed (5-5’, 4-*O*-5’) units [21]. Patterns occurring at +18 amu after every principal peak account for the formation of β-*O*-4 bonds, which is subjected to water insertion on the intermediate quinone methide.

**Figure 6.**
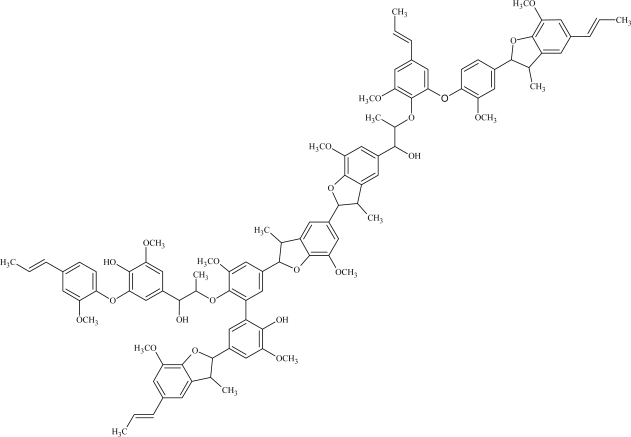
Representative “lignin-like” product of isoeugenol polymerization.

Altogether, these results are in agreement with a radical coupling mechanism. The reaction starts with the formation of phenoxy radicals by metal catalyst-mediated H abstraction. Then, the mesomeric delocalization on the phenylpropenoid structure lead to various type of radical coupling reactions. The resulting oligomers are characterized by different intermonomeric linkages, mainly represented by β-*O*-4 and β-5 bonds [25,15]. On the basis of NMR, GPC and LC-MS qualitative and quantitative data it was possible to attribute a tentative formula, accounting for a ‘lignin-like’ structure, to the isoeugenol DHP (Figure 6). A representative DHP oligomer was assumed to contain about 10 repeating units, connected by β-5, β-*O*-4 bonds and condensed units (5-5’, 4-*O*-5’) as the principal intermonomeric linkages.

### 2.4. Radical Scavenging Activity

The obtained DHP is proved to possess valuable antioxidant and antibacterial activity. Elegir and coworkers studied the antibacterial properties of lignocellulosic fibers treated with isoeugenol and laccase as grafting initiator [17]. The antibacterial activity *versus Staphylococcus aureus* was strongly enhanced by the polymerization with respect to the isoeugenol monomer. This important property could be applied in the development of antimicrobial packaging based on lignocellulosic materials. The main mechanism involved in the antioxidant activity of phenolic compounds is supposed to be the scavenging of free radicals. Therefore, the reactivity towards the stable radical DPPH• of both isoeugenol and DHP was tested (Table 6).

Isoeugenol showed a higher radical scavenging activity, with an effective concentration of DPPH• radical (IC_50_) equal to 0.195 mg[AH]/mg[DPPH•]. Alternatively, this result could be expressed in mole (0.48 mol per mol of DPPH•) and it is in agreement with literature data [21,26]. The data evidenced that the molar ratio isoeugenol/DPPH• needed for a complete reaction is 1.04, indicating that one mole of phenolic groups reacts with one mole of DPPH•. When the DHP is concerned, the observed radical scavenging activity show a higher IC_50_ value, 1.41 mg per mg of DPPH. However, the data is in agreement with the total phenolic content of the DHP (1.1 mmol/g), demonstrating that the radical scavenging activity of the isoeugenol DHP is also related to the amount of phenolic groups.

## 3. Experimental Section

### 3.1. Materials

Isoeugenol (99%, mixture of cis and trans), ethanol (99.9%) and ethyl acetate (99.9%) were purchased from Fluka. The bulky salen complexes was synthesized according to literature methods [6]. TLC was performed on precoated Merck Silica gel 60 F_254_ plates and visualized under UV light.

### 3.2. General Procedure for Oxidative Coupling of Isoeugenol

Isoeugenol (w/v%) and the catalyst (w/v%) was dissolved in a 1:1 mixture of ethanol/water (10 mL). Then H_2_O_2_ (mol/mol respect to isoeugenol) was added at different rates or the mixture was stirred under a dioxygen atmosphere. The reaction was monitored by TLC (2:1 petroleum ether-ethyl acetate as the eluent). After about 48 hours the mixture was diluted with water (15 mL) and extracted into ethyl acetate (3 × 15 mL). The extract was washed with brine (20 mL), dried over NaSO_4_, filtered and the solvent was then evaporated under reduced pressures. The polymerization yield, evaluated as weight percentage, was typically ≥ 90%.

### 3.3. Gas Chromatography-Mass Spectrometry (GC-MS)

Isoeugenol oxidation products were extracted in ethyl acetate and purified by separation on a silica gel column. The solvent was evaporated under reduced pressure. The crude was derivatized with 1 mL of *N,O*-bis(trimethylsilyl)trifluoroacetamide (1% trimethylchlorosilane). The solvent was removed and the derivatized sample dissolved in CH_2_Cl_2_ (1 mg/mL). Two μL of the solution were then injected into a GC/MS spectrometer equipped with a 30 m Supelco SPB-5 (95% dimethylpolisiloxane) capillary column (inner diameter 0.25 mm, film thickness 0.25 μm). The column was eluted at 80 °C for 4 min, followed by a temperature gradient from 80 °C to 220 °C at 10 °C/min and then from 220 °C to 280 °C at 8 °C/min. The carrier gas was helium at a flow rate of 40 cm/s.

### 3.4. Gel Permeation Chromatography (GPC)

GPC analyses were performed on an Agilent 1000 liquid chromatograph interfaced to a diode array (UV) detector. The column was an Agilent PL 3 μm MIXED gel E MW 220–400 W and the solvent used was tetrahydrofuran for HPLC (>99.8%). Polystyrene standards were used for calibration. Evaluation of number-average molecular weight (M_n_) and weight-average molecular weight (M_w_) was carried out following published methods [27,28].

### 3.5. Statistical Analyses of Data

The use of factorial analysis of variance (ANOVA) was made to determine the effects of the various experimental variables (oxidant concentration, number of oxidant aliquots) examined in this work on the average molecular weight of the obtained polymer (XLSTAT 2008 software).

### 3.6. ^13^C-NMR and ^31^P-NMR Studies

Reaction products were acetylated in 1:1 v/v acetic anhydride-pyridine solution and subjected to quantitative ^13^C-NMR analysis, as reported by Canevali *et al*. [20]. ^31^P-NMR of a suitably derivatized sample was performed in order to characterize and quantify different functional groups with labile -OH. In order to perform phosphorus analysis, the sample was derivatized with 2-chloro-4,4,5,5-tetramethyl-1,3,2-dioxaphospholane as reported in literature [22]. The ^31^P-NMR data reported in this article are averages of three experiments. The maximum standard deviation of the reported data was 2 × 10^−2^ mmol/g, while the maximum standard error was 1 × 10^−2^ mmol/g.

### 3.7. LC-MS

LC-MS analysis was performed on the crude polymer by means of a Shimadzu HPLC coupled to a Shimadzu 2010 mass spectrometer by an APCI interface. The sample was dissolved and diluted in methanol until a concentration of 10 ppm was reached. Then, 5 μL of the previous solution were analyzed by direct-injection. The analysis were performed in positive scan, using methanol/water 1:1 as the eluent at a flow rate of 1 mL/min. The carrier gas was molecular nitrogen set at a flow rate of 1.5 mL/min. In the APCI ion source the temperature was fixed at 200 °C and the voltage set to +4.5 kV. The mass acquisition range was comprised within 100 and 2,000 amu.

### 3.8. Estimation of Radical Scavenging Activity

The radical scavenging activity of isoeugenol and the corresponding DHP was determined by means of a spectroscopic assay involving the consumption of the stable free radical originated by DPPH in an ethanol solution (0.1 mM). Reduction in DPPH• concentration was monitored by absorbance measurement at 516 nm. Different concentrations, expressed as mg of antioxidant [AH] per mg of [DPPH•], were tested. From the resulting graphs it was possible to estimate the percentage of unreacted [DPPH•] at the steady state. These values were then transferred onto a graph showing the percentage of residual stable radical at the steady state as a function of the weight ratio [AH]/[DPPH•]. The latter ratio was used to determine the efficient concentration (IC_50_), indicating the amount of antioxidant needed to halve the initial DPPH• concentration.

## 4. Conclusions

Water soluble salen complexes catalyze the polymerization of isoeugenol through dehydrogenative radical coupling in a water-ethanol solution and the molecular weight of the obtained DHP is strictly related to the reaction conditions. The best result was accomplished using CuSS as catalyst and hydrogen peroxide as oxidant. The chemical structure of the isoeugenol polymer is comparable with DHPs obtained from the polymerization of coniferyl alcohol. The radical scavenging activity of the isoeugenol DHP is lower than that of isoeugenol monomer but, nevertheless, in agreement with the amount of phenolic groups.

## Figures and Tables

**Table 1.** Results of GPC analyses on oxidation products of isoeugenol with different catalyst.

**Catalyst**	**Oxidant**	**M_n_**	**M_w_**	**M_w_/M_n_**
CuSS	O_2_	850	1260	1.48
CuSS	H_2_O_2_	1230	2840	2.30
CoSS	O_2_	530	830	1.56
MnSS	H_2_O_2_	510	620	1.22
FeSS	H_2_O_2_	830	1350	1.62
CuPS	O_2_	480	750	1.56
CuPS	H_2_O_2_	925	1465	1.22
CoPS	O_2_	380	505	1.36

**Table 2.** Results of GPC analyses on the oxidation products of isoeugenol with CuSS at different pH.

**pH**	**M_n_**	**M_w_**	**M_w_/M_n_**
4.5^a^	843	1222	1.45
7^b^	936	1537	1.82
9^c^	1013	1748	1.72

a buffer solution 0.1 M Citric acid/Citrate.

b buffer solution 0.1 M NaH_2_PO_4_/Na_2_HPO_4_.

c buffer solution 0.1 M NaHCO_3_/Na_2_CO_3_.

**Table 3.** Results of GPC analyses on oxidation products of isoeugenol applying Zutropf (ZT) and Zuluaf (ZL) conditions.

**Method**	**M_n_**	**M_w_**	**M_w_/M_n_**
ZT	1230	3108	2.52
ZL	1076	2675	2.48

**Table 4.** Results of GPC analyses on coupling products of isoeugenol with CuSS at different concentrations of catalyst and monomer.

**Isoeugenol w/v%**	**Catalyst w/v%**	**M_n_**	**M_w_**	**M_w_/M_n_**
1	0.02	832	1487	1.79
1	0.05	981	1698	1.73
1	0.1	1190	2157	1.81
1	0.2	1522	3652	2.40
0.5	0.1	570	1100	1.92
1	0.1	1230	2840	2.30
5	0.1	1530	3390	2.21
10	0.1	1270	3010	2.37

**Table 5.** Results of GPC analyses on coupling products of isoeugenol in the presence of CuSS as catalyst when the oxidant concentration and the oxidant addition rate were varied.

**Oxidant Concentration (mol H_2_O_2_/mol isoeugenol)**	**Number of H_2_O_2_ aliquots**	**M_n_**	**M_w_**	**M_w_/M_n_**
5	1	2030	7600	3.75
5	4	1690	5570	3.31
5	8	1260	2650	2.10
5	16	1450	3500	2.41
10	1	1230	2840	2.30
10	4	2440	8460	3.46
10	8	1950	7760	3.98
10	16	1480	3250	2.19
15	1	1330	2700	2.03
15	4	1440	3260	2.19
15	8	1630	3430	2.11
15	16	1670	4120	2.47

**Table 6.** Scavenging activity of isoeugenol and DHP toward DPPH.

**Phenol (AH)**	**IC_50_ (mg of AH/mg of DPPH♦)**
isoeugenol	0.195
DHP	1.41
